# Notes and News

**Published:** 1898-07-16

**Authors:** 


### Notes and News.

(Collected and Communicated.)

The *Medical News* of New York says that the Maryland State Legislature has appropriated 400,000 dollars to the Johns Hopkins University to tide it over its financial straits, due to the loss sustained in the default of the Baltimore and Ohio Railroad.

Universal admiration has been expressed for the prompt assistance and admirable organisation shown by the medical and nursing staffs of the Poplar Hospital on the occasion of the disaster at the Blackwall launch. Within a few minutes of the notification of the accident 45 beds were in absolute readiness.

At the annual meeting of the governors of the National Society for Employment of Epileptics held on July 11th, it was stated that a marked improvement had been noted in the majority of cases at the Chalfont Colony, this improvement being especialy pronounced in the case of the women colonists. Besides one home for 24 colonists, recently completed but not yet occupied, four others are now in course of erection, the cost having been provided by contributions for this specific purpose. The number of candidates for admission continues to increase, and H.R.H. the Duke of York, as president, is issuing a special appeal on behalf of the society for the sum of £7,000 for certain immediately necessary purposes detailed in the report.

It is impossible to approve of the dramatic and altogether objectionable custom, which appears to be a growing one, at some local and provincial hospital collecting days of setting up cots in the public streets containing sick children, whose infirmities are thus displayed in the highways and byeways as an incentive to passing charity. The drawing of invalid chairs about the streets with pathetic sick freights is another development of a theatrical and charitable "playing to the gallery" which should be strongly discountenanced by the public. Hospital cycling parades and demonstrations are perfectly legitimate, but when such "demonstrations" include the travesty of suffering made by trading on infirmity, it is time some protest were offered.

The rite of "kissing the book" has been abolished in Maryland, and the form of the judicial oath has been simplified.

Mr. M. Garton, a well-known brewer, has generously offered to complete, at a cost of £5,000, the new wing of the South Hants Infirmary, a work which it was hoped would be carried out as a Jubilee celebration, but failed of completion owing to the public funds falling far short of the sum needed for the project.

An interesting old building in Newcastle is likely to be soon pulled down from sheer old age. In 1700 the Tyne keelmen petitioned the Council for a grant of land to erect a hospital. Each keelman contributed 4d. per tide to the institution, for which the ground rent of 6d. per half-year is paid. The institution was for many years tenanted by keelmen and their belongings, but the "keelman" has more or less disappeared, the keel having vanished before steam tugs and similar craft. Some of the inmates of the institution have lived there for upwards of sixty years, and are broken-hearted at the prospect of their "home" being pulled down.

On July 7th a new wing was added to the hospital at Rotherham, Yorkshire, for the reception of sick children, and formally opened by Lord Scarborough. The ward, which was built in commemoration of Her Majesty's Diamond Jubilee, is named Queen's Ward, the stone being laid by the Mayor of Rotherham (Alderman Neill, J.P.) on Jubilee Day last year. Accommodation is provided for 14 cots in the large ward, and two in an isolation block, which has its own separate arrangements. The floors are of terrazzo, the walls of Parian cement, the dado and large stove being fitted with Burmantoft tiles. The lavatory and bathroom fittings are on the latest sanitary principles. A new operating theatre is in course of erection, and new bed-rooms are shortly to be added for the increasing staff of nurses.

At the last meeting of the Committee on the Vaccination Bill, a curious clause was proposed by Mr. Steadman, providing that in all cases of severe illness following vaccination the operating surgeon should be liable to an action for damages as in any other case of malpractice, and that in case of death the surgeon should take his trial for manslaughter. This amendment was by leave withdrawn, but the very fact that it was proposed shows how vague are the notions of some members of Parliament as to the duties and responsibilities of the surgeon, and the causes of non-success or of the evil consequences which may occasionally follow vaccination. As a fact, no such clause is necessary even from the point of view of those who would put every obstacle in the way of vaccination, for if a surgeon can be shown to have displayed gross negligence, or ignorance, or want of care in the treatment of a case of vaccination, he is subject to the law just as he is in regard to any other operation, and, indeed, he is far more liable to be brought to book than in regard to ordinary cases, for, whereas many a death after operation is buried on a mere certificate, custom has rendered an inquest almost obligatory in the case of death occurring soon after vaccination.

